# Adenosquamous Carcinoma of Gallbladder: A Rare Case Report

**DOI:** 10.7759/cureus.68049

**Published:** 2024-08-28

**Authors:** Bibhusha Khanal, Prakriti Lamichhane, Ranjan R Bhatta, Shankar Bastakoti

**Affiliations:** 1 Pathology, Lumbini Medical College, Palpa, NPL; 2 Pathology, KIST Medical College, Lalitpur, NPL; 3 Pathology, BP Koirala Memorial Cancer Hospital, Bharatpur, NPL

**Keywords:** extended cholecystectomy, carcinoma, gall bladder, adenocarcinoma, adenosquamous carcinoma

## Abstract

Carcinoma of the gallbladder is an uncommon malignancy with a poor prognosis overall. Histologically, adenocarcinoma is the most common type of gallbladder carcinoma. Adenosquamous carcinoma is a rare histological type of gallbladder carcinoma comprising both the glandular and squamous elements. Adenosquamous carcinoma shows more aggressive behavior than adenocarcinomas and is often detected in a late advanced stage. Treatment is usually extended surgical resection but has a poor prognosis. We present a rare case of adenosquamous carcinoma with lymphovascular invasion in a 72-year-old male who was managed with extended cholecystectomy.

## Introduction

Gallbladder (GB) carcinoma is an uncommon malignancy, however, in the biliary tract, it is the most common malignancy [1,2]. The most common histological subtype is adenocarcinoma, accounting for approximately 90-95% of all malignancies while adenosquamous cell carcinoma (ASC) is extremely uncommon, accounting for about 5% of cases only [2-5]. There is a considerable debate about its histogenesis whether there is squamous differentiation in an adenocarcinoma or it is a neoplastic process of squamous cell carcinoma (SCC) [6]. ASCs show aggressive biological behavior regardless of how they arise due to their capacity for direct extension into the adjacent viscera and early metastasis [6-8]. They lack specific clinical presentation until the growths become substantially large and at an advanced stage [7].

## Case presentation

A 72-year-old mongoloid male presented to the primary physician with complaints of right upper quadrant abdominal pain, colicky in nature, gradual in onset, and progressively worse over the last two months. He took paracetamol daily with some pain relief. He also complained of occasional heartburn relieved by over-the-counter proton pump inhibitor (PPI). He denied any changes in weight or changes in bowel habits. He's an ex-smoker, smoked for 10 years, and stopped smoking and drinking alcohol for the last six months. He has a history of pulmonary tuberculosis 10 years back and completed treatment as per the national guidelines with antitubercular therapy (ATT) for six months. On physical examination, his vitals were stable and other systemic examinations including his abdomen were also normal. Laboratory investigations showed moderate thrombocytopenia (71,000/mm^3^), normal liver function test, and normal tumor markers including CA19-9. Ultrasonogram (USG) of the abdomen revealed minimal echogenic sludge in the GB lumen. Upper gastrointestinal endoscopy (UGIE) revealed Grade-I esophageal varices with hiatal hernia, antral, and fundal ulcer with erosive pangastritis. Contrast-enhanced tomogram (CECT) of the abdomen later showed a distended GB with soft tissue thickening measuring a maximum of 13mm in thickness and 50mm in transverse length along the lateral wall of GB. The soft tissue thickening was seen to have a loss of fat planes with the adjacent liver parenchyma (segment VI of the right lobe) in the USG. Imaging findings suspicious for neoplasm.

The patient underwent extended cholecystectomy. Intra-operatively, a mass was noted in the body of GB along the posterior wall with invasion to segments IVa & b of the liver, and multiple enlarged lymph nodes along segments VI, VII, and VIII. The specimens of the GB with liver margin and lymph nodes were sent for histopathological examination in formalin.

**Figure 1:**
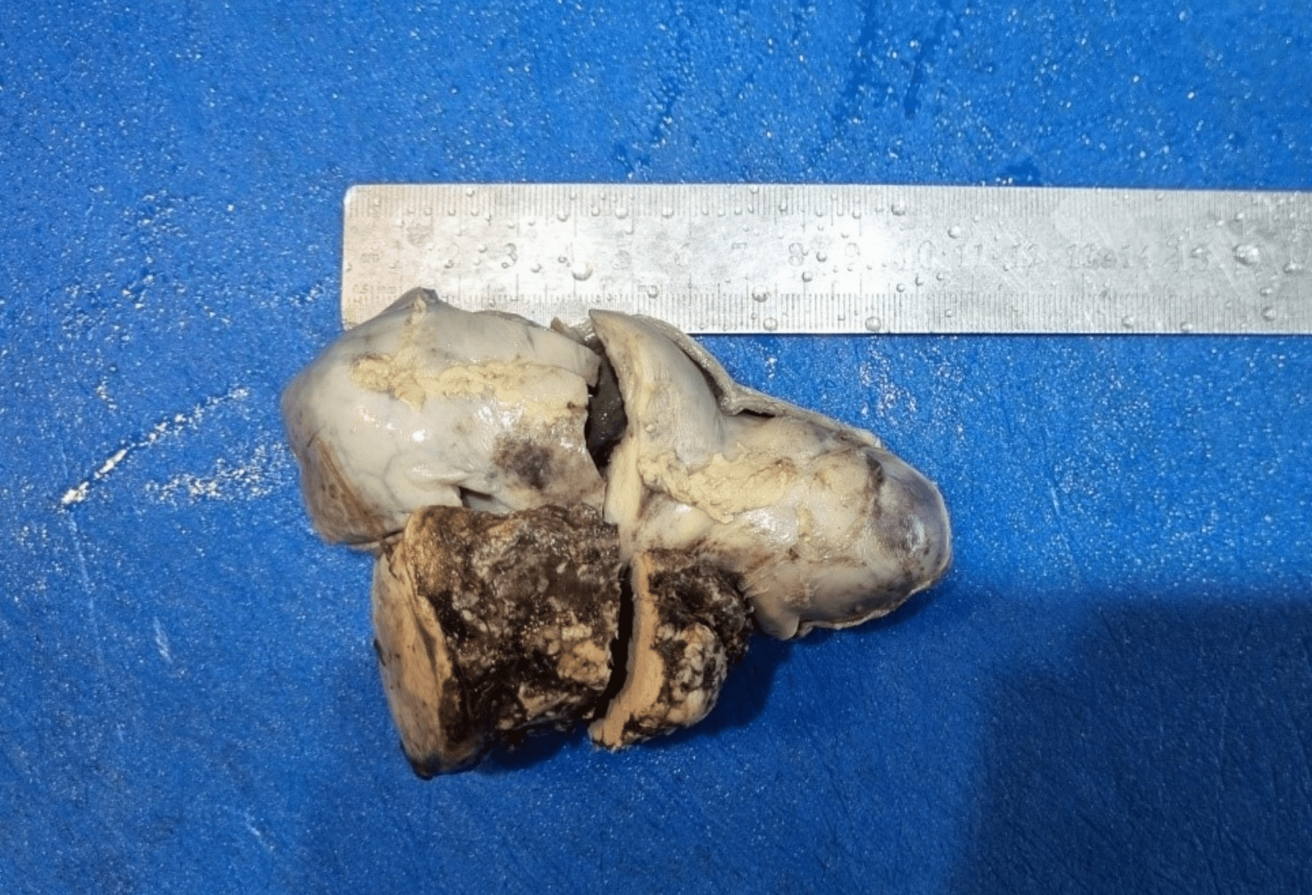
Gross specimen of the gallbladder mass

**Figure 2:**
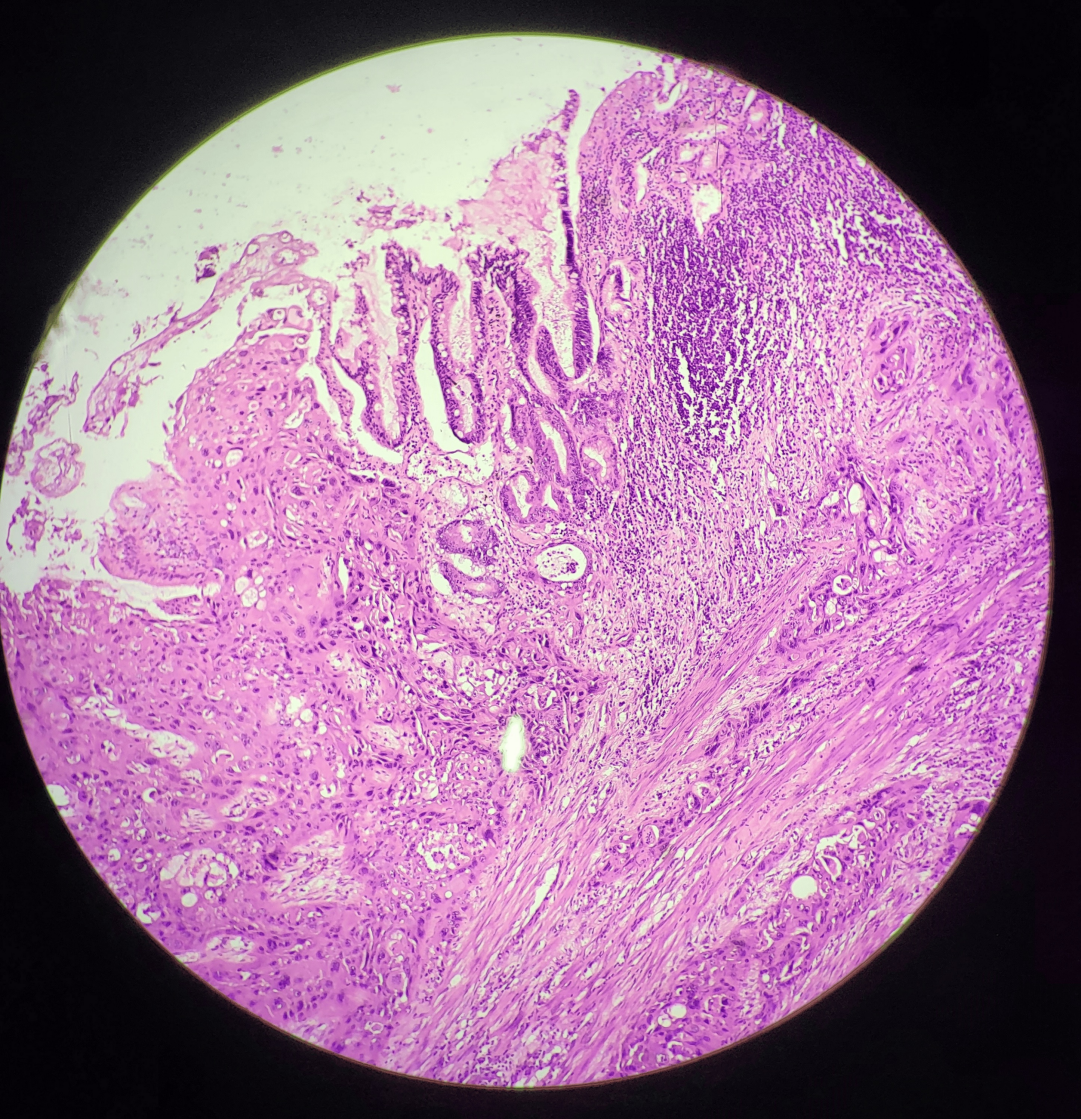
Histopathological section showing malignant cellular changes and invasion, confirming the adenosquamous carcinoma of gallbladder

**Figure 3:**
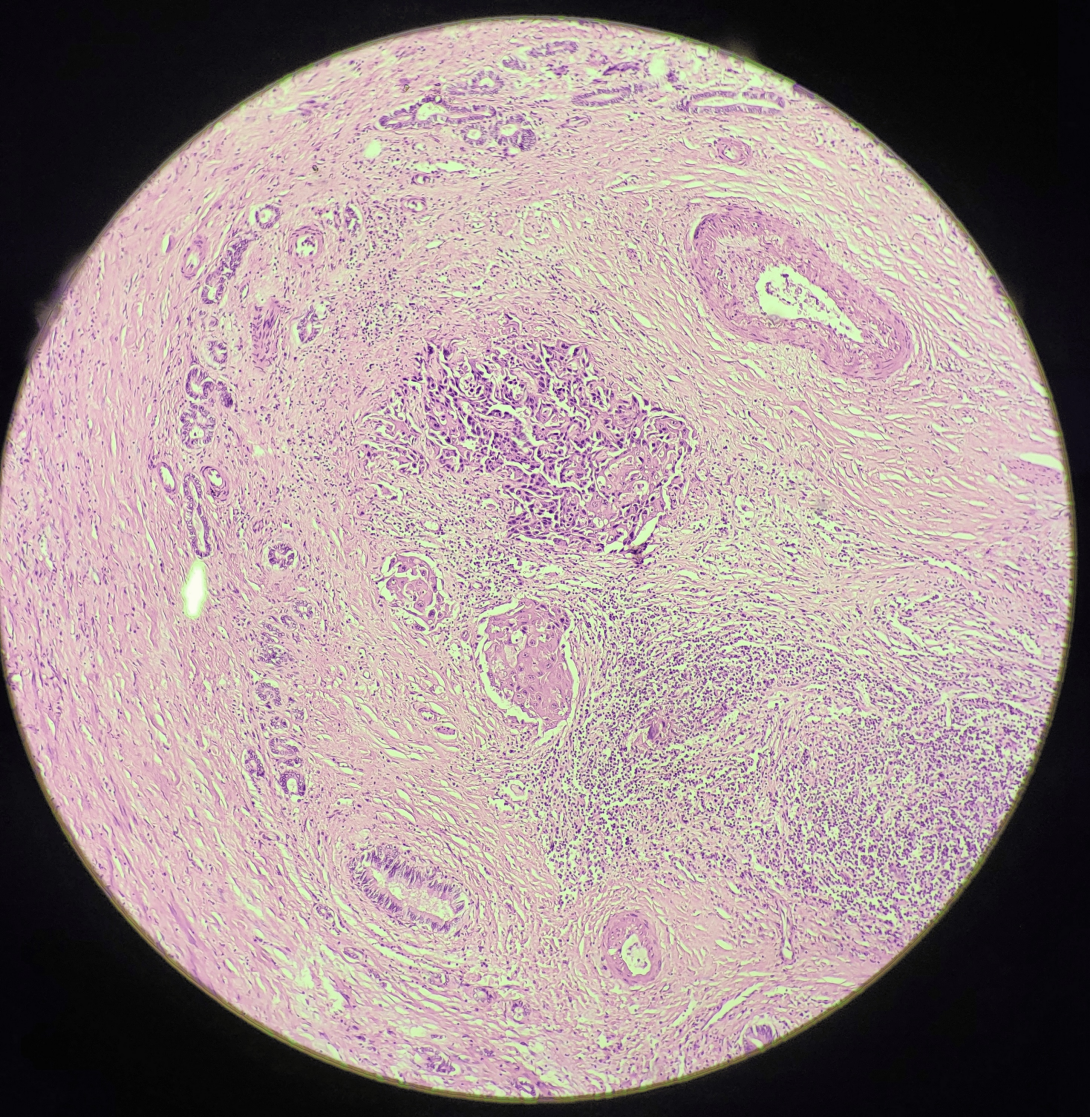
Histopathological section showing adenosquamous components and mitoses

**Figure 4:**
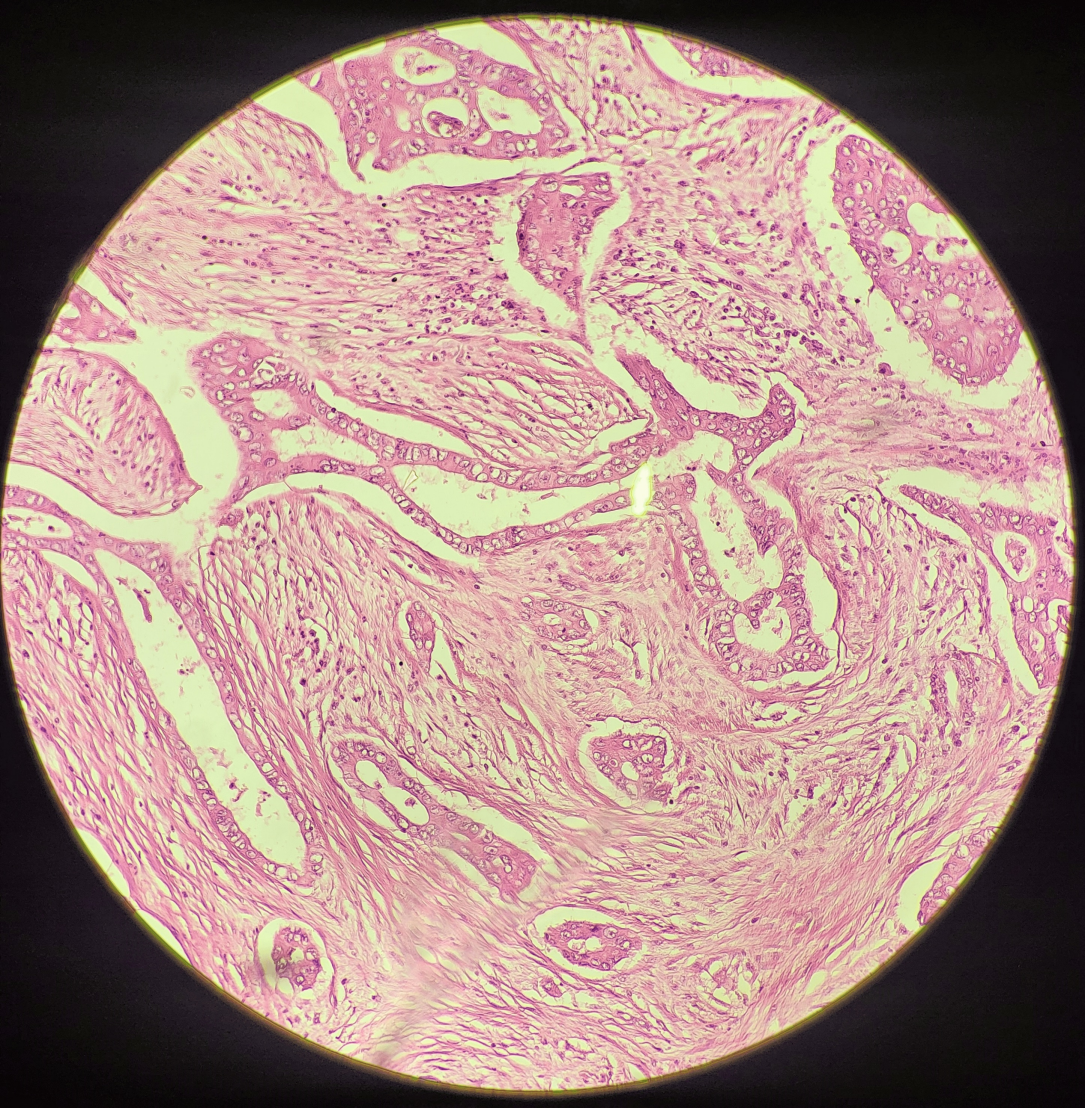
Histopathological section showing the glandular components (higher magnification)

**Figure 5:**
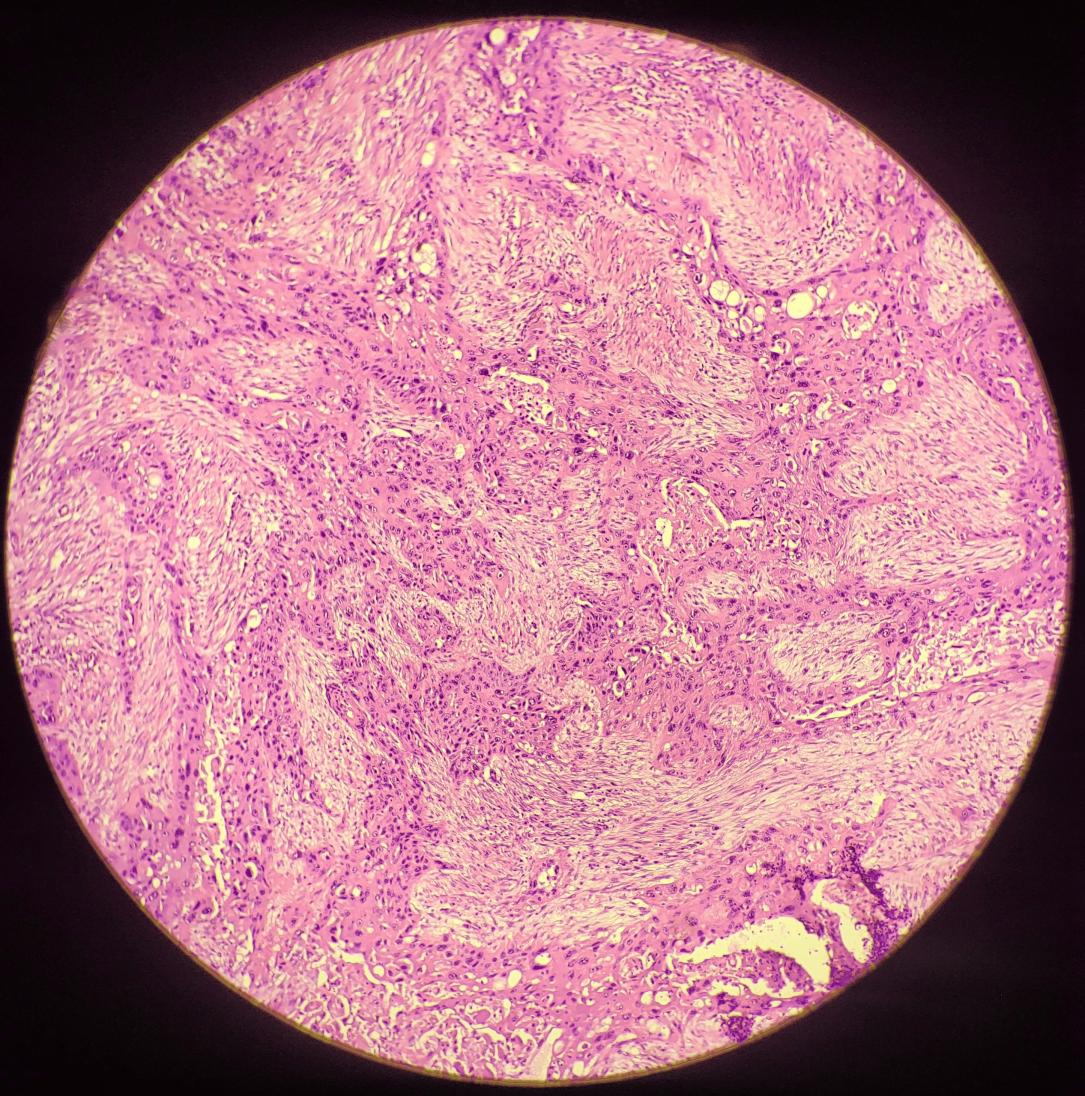
Histopathological section showing the squamous components (higher magnification)

On gross pathological examination, the GB measured 7x5 cm (Figure 1). The cut section revealed a mass measuring 4x2.5 cm involving the liver, along with a yellow-appearing stone measuring 2x1 cm. On microscopic examination (Figures 2-5), tumor cells were arranged in nests, clusters, and sheets showing pleomorphism, anisokaryosis, karyomegaly, high nuclear to cytoplasmic ratio, irregular nuclear rim, hyperchromatic to vesicular chromatin, prominent nucleoli, and moderate eosinophilic cytoplasm. Individual cell keratinization and intercellular bridges were also seen. Neoplastic mucosal glands were seen invading up to and beyond the muscle layer. Mitoses including atypical mitotic figures were noted. Both the adenocarcinoma and squamous cell carcinoma components were intermingled with few areas showing abrupt transition. Lymphovascular invasion was evident.

## Discussion

Epithelial malignancies of GB (carcinomas) are rare and have geographical and ethnic variations. The highest incidence of GB carcinoma is seen in Chile (27.3 cases per 1,00,000 person-years) among the indigenous Mapuche people, predominantly in females [9]. India, eastern Asia, and central and eastern European countries also show high incidence [3-5,10,11]. Known risk factors for the development of GB cancer include female gender, obesity, diabetes, gallstones, chronic cholecystitis, primary sclerosing cholangitis, certain gallbladder polyps, and porcelain gallbladder [4,11].

GB cancers are histopathologically classified into adenocarcinoma, squamous cell carcinoma (SCC), or adenosquamous carcinoma (ASC) [4,5]. Adenocarcinoma is the most common subtype, making up around 90-95% of all cases [2,4]. ASC is a much less common histopathological subtype accounting for approximately 5% of the cases [4,5,11]. According to WHO, if squamous elements constitute a substantial part of the neoplasm (>25%), it is best classified as ASC [2,5]. Some believe that the risk factors of adenocarcinoma and ASC are different, however, a conclusive opinion was not possible due to the small number of cases in the studies [2,4,12]. One of the studies involving 34 cases of ASC found female predominance, thus a potential risk factor [2].

GB carcinoma often presents late and at an advanced stage and, therefore, has a poor prognosis with a five-year survival rate of less than 5% [4,13]. Patients with ASCs have a worse prognosis than those with stage-matched advanced adenocarcinoma [2,9]. One study of 606 GB carcinomas, including 34 ASCs showed a median survival time of 4 months vs 11 months in adenocarcinoma [2]. Some other studies suggest ASC has an aggressive behavior probably due to its possible histogenesis from metaplastic changes, owing to its high proliferative capacity in the GB [2,9]. Direct extension to the liver during surgical resection was also noted in other studies. Although found normal in our case, tumor markers CEA and CA19-9 play a vital role in the diagnosis and prognosis of oncogenesis [4,9,11,14]. CA19-9 may be a helpful indicator for prognosis rather than the gold standard as cases of acute cholecystitis or pancreatitis may also present with increased CA19-9 levels [15].

The curative treatment of GB cancer is surgical removal of the tumor with negative surgical resection margins. Resection of the hepatic bed of GB and lymph node dissection (at least six lymph nodes) is also necessary but the main bile duct resection is not mandatory in the absence of invasion of the cystic duct or the main bile duct [16].

## Conclusions

Adenosquamous carcinoma of GB is a rare GB malignancy and is often detected late with extension to adjacent viscera and metastasis. It is more aggressive and has a poor prognosis than GB adenocarcinoma. Early detection and a multidisciplinary approach to care can help with better survival. Extensive studies are warranted to understand this uncommon malignancy and its optimal management.
